# Hantavirus Reservoirs: Current Status with an Emphasis on Data from Brazil

**DOI:** 10.3390/v6051929

**Published:** 2014-04-29

**Authors:** Renata Carvalho de Oliveira, Alexandro Guterres, Jorlan Fernandes, Paulo Sérgio D’Andrea, Cibele Rodrigues Bonvicino, Elba Regina Sampaio de Lemos

**Affiliations:** 1 Laboratório de Hantaviroses e Rickettsioses, Instituto Oswaldo Cruz, Fundação Oswaldo Cruz, Rio de Janeiro, 21040-360, RJ, Brazil; E-Mails: guterres@ioc.fiocruz.br (A.G.); jorlan@ioc.fiocruz.br (J.F.); elemos@ioc.fiocruz.br (E.R.S.L.); 2 Laboratório de Biologia e Parasitologia de Mamíferos Silvestres Reservatórios, Instituto Oswaldo Cruz, Fundação Oswaldo Cruz, Rio de Janeiro, 21040-360, RJ, Brazil; E-Mails: dandrea@ioc.fiocruz.br (P.S.D.); cibelerb@inca.gov.br (C.R.B.); 3 Programa de Genética, Instituto Nacional de Câncer, Ministério da Saúde, Rio de Janeiro, 20231-050, RJ, Brazil

**Keywords:** hantavirus, reservoirs, hantavirus pulmonary syndrome, haemorrhagic fever with renal syndrome

## Abstract

Since the recognition of hantavirus as the agent responsible for haemorrhagic fever in Eurasia in the 1970s and, 20 years later, the descovery of hantavirus pulmonary syndrome in the Americas, the genus *Hantavirus* has been continually described throughout the World in a variety of wild animals. The diversity of wild animals infected with hantaviruses has only recently come into focus as a result of expanded wildlife studies. The known reservoirs are more than 80, belonging to 51 species of rodents, 7 bats (order Chiroptera) and 20 shrews and moles (order Soricomorpha). More than 80genetically related viruses have been classified within *Hantavirus* genus; 25 recognized as human pathogens responsible for a large spectrum of diseases in the Old and New World. In Brazil, where the diversity of mammals and especially rodents is considered one of the largest in the world, 9 hantavirus genotypes have been identified in 12 rodent species belonging to the genus *Akodon*, *Calomys*, *Holochilus*, *Oligoryzomys*, *Oxymycterus*, *Necromys* and *Rattus*. Considering the increasing number of animals that have been implicated as reservoirs of different hantaviruses, the understanding of this diversity is important for evaluating the risk of distinct hantavirus species as human pathogens.

## 1. Introduction

The genus *Hantavirus*, which consists of more than 80 genetically related viruses, was first identified as an agent of the human disease haemorrhagic fever with renal syndrome (HFRS) in the 1970s [1,2]. After characterization of the Hantaan virus prototype in samples from humans and the rodent reservoir *Apodemus agrarius*, other species linked to HFRS in Eurasia, such as Pummala, Seoul and Dobrava-Belgrado, were identified. The Dobrava-Belgrado species is thought to be responsible for the majority of deaths from hantaviruses in the Old World [3,4,5].

Hantavirus has been known to circulate throughout the Americas in the rodents *Microtus pennsylvanicus* (virus Prospect Hill) and *Rattus novegicus* (virus Seoul) since the 1980s. In 1993, the novel hantavirus Sin Nombre was identified, and researchers determined that infection by the genus *Hantavirus* causes hantavirus pulmonary syndrome (HPS) or hantavirus cardiopulmonary syndrome (HCPS), which is restricted to the Americas [6,7,8,9].

Since HPS was identified in the United States, the number of new hantaviruses discovered in rodent reservoirs and more recently in insectivorous and bats has grown steadily. Hantaviruses are considered infectious agents of great importance for public health and are broadly distributed around the globe. Hantaviruses are trisegmented RNA that belong to the genus *Hantavirus* and the family *Bunyaviridae*. Humans acquire HPS/HCPS and HFRS mainly by inhalation of viral particles present in aerosols that arise from rodent excretions and secretions [10,11].

Hantaviruses are distributed focally because transmission depends critically on the rodent reservoir in addition to human activity. With various clinical-epidemiological patterns depending on the human behavior, the natural history and the distribution of their reservoirs, the hantaviruses have been identified worldwide. In Asia and Europe, the hantaviruses that cause HFRS are responsible for approximately 150,000 to 200,000 cases per year with mortality rates ranging from <1% to 12%. There are approximately 200 cases of HPS per year in South America, with a mortality rate around 40% [12,13].

According to the International committee on the taxonomy of viruses (ICTV), four criteria are used to classification hantavirus species [14]. Thus, a virus under study is considered a novel hantavirus species when the following criteria are met: (i) belongs to a unique ecological niche, such as targeting a distinct rodent species or subspecies as the primary reservoir; (ii) exhibits at least 7% difference in the amino acid sequence of surface glycoproteins (Gn and Gc) of the viral nucleoprotein; (iii) shows at least a four-fold difference in the antibody titre in cross-neutralization tests; and (iv) does not form natural rearrangements with other hantavirus species [15]. Of the various hantaviruses described worldwide, 24 have been officially accepted and classified as species by the ICTV [16,17].

Referred to by different authors as species, variants, genotypes, strains, lineages or serotypes, despite the four species-defining criteria established by the ICTV the continuing identification of hantaviruses exhibits a lack of agreement with respect to naming terminology. Firth *et al*. recently suggested that newly identified hantaviruses that do not meet the species criteria and are related to other pre-existing viruses should be classified as genotypes according to a previously established standard, whereby the abbreviated hantavirus name is followed sequentially by a number [18].

With the on-going identification of novel hantaviruses in rodents that may or may not be associated with human disease, these viral agents will be recognized together with arenaviruses as the rodent-borne “Robo” viruses [19]. However, nearly 40 years after the initial description of the Thottapalayam (TPM) hantavirus, which was isolated in 1964 [20] from an Asian house shrew species (*Suncus murinus*) captured in India and classified as a member of the genus in 1989 [21], novel hantaviruses that are genetically distant from the hantaviruses associated with rodents were identified in other vertebrate species, leading to the adoption of terms such as “InBo-viruses” and “RaInBo-viruses” [22,23,24,25,26,27,28].

Despite the identification of novel hantaviruses in samples from human cases, rodents, insectivores (families Sociridae and Talpidae) and bats (families Vespertilionidae and Nycteridae) that are not associated with human disease, the information obtained to date is insufficient to understand the numerous particularities with respect to the interactions between hantaviruses and their vertebrate reservoirs during the transmission cycles that occur in the wild [29,30,31].

Given the importance of correct taxonomic identification of reservoirs for a complete understanding of hantavirus transmission cycles and for assessing their potential as host transmitters of hantavirus to humans, certain factors that increase our understanding of the virus-host interaction dynamics and the risk of human infection must be identified. These factors include the following:

(i) geographical distribution of the host to map the maximum area where the hantavirus species is distributed and, consequently, where the hantavirus species that colonizes this host may occur;
(ii) abundance of the reservoir species in the natural environment, which is considered a strong indicator of greater risk for human infection based on the fact that hantaviruses are zoonosis whose transmission depends on host population density;
(iii) how the agent interacts with the host reservoir, which is a critical factor for understanding the spread and maintenance of hantaviruses in the environment. Lack of illness in the reservoir and the establishment of prolonged infection that may persist for the entire lifetime of the reservoir animal ensure that the hantavirus will remain in the environment throughout the lifespan of the reservoir animals and ensures transmission among rodents and;
(iv) host/reservoir habits in natural and anthropic environments. For example, the tendency of rodents to enter homes is an important factor in hantavirus epidemiology because this occurrence allows these reservoirs to come into close contact with humans.

Despite the issues stated above, other factors associated with the process of anthropisation are related to the risk of hantavirus transmission. These factors include disruption of rodent habitats, which can be caused by deforestation and extensive agriculture and can favor opportunist or generalist species that can serve as hantavirus reservoirs. Environmental changes that reduce rodent diversity is another important factor because environmental changes permit aggressive encounters between animals, thereby increasing interactions between host species that can trigger a cascade of greater viral transmission within a single species or across different rodent species and can lead to greater risk of spillover (transfer of hantavirus from one rodent species to another) in rodents. Depending on their behavior, these species could develop more frequent contact with human populations [32,33].

In this context and given the growing number of publications over the past two decades on hantaviruses in wild reservoirs, this review addresses relevant aspects of the global distribution of hantavirus reservoirs, with an emphasis on the Americas—particularly Brazil, and provides an update on the information available in the scientific literature. Additionally, based on the principle that geographic distribution of host(s) defines the maximum area where illness can be endemic and based on the fact that, in contrast to North America, the distributions of most small mammalian species are well known, in this review, the potential area for each viral genotype recognized in South and Central America is determined based on the known distribution of its hosts [34,35,36].

## 2. Hantavirus Hosts

### 2.1. Rodents

There are nearly 2200 rodent species worldwide, which represent approximately 42% of all mammalian species [37]. Rodents exhibit an extraordinary variety of ecological and physiological adaptations to almost all terrestrial environments; it can live in high elevations, extreme climates, regions with extensive vegetative cover and regions that are barren.

Some species are considered synanthropic because they associate with humans. In urban and rural environments with economic activity, commensal synanthropic species are predominant, as well as some wild species that occasionally invade human dwellings.

The rodent species described as reservoirs of hantaviruses are classified into two families: family Muridae, subfamily Murinae (rats and mice), which were found in the Old World; and family Cricetidae, which is divided into the three subfamilies: Arvicolinae (voles and lemmings found in Eurasia and North America), Neotominae and Sigmodontinae (rats and mice found in the New World) [37].

Although each hantavirus is predominantly associated with a specific rodent and a particular location, infections in other hosts by the same virus can occur by “spillover” or interspecific transmission. Spillover events occasionally occur in nature within a geographical region and have been documented for various viruses and sympatric (species occurring in the same geographic region) or syntopic (species sharing the same habitat within the same geographical range) rodent host species, thus providing more evidence that heterologous (secondary) host species are susceptible to infection [9,19,38]. Moreover, multiple host models have indicated that under certain circumstances, persistence of infection within rodent populations can increase with the presence of secondary hosts and spillover [39,40]. Environmental changes that result in greater habitat overlap can create more interspecific encounters that could lead to outbreaks or even the establishment of the viral colonization of a novel host [41]. However, the virus-host relationship generally appears stable, even if hantaviruses are sometimes found in other hosts. “Host-switching” events can be observed in phylogenetic trees when the topologies of the host and virus do not coincide [42,43,44].

#### 2.1.1. Taxonomical Characterization

Given the close link between hantaviruses and their rodent hosts, the main question to be addressed when studying hantavirus infections in rodents is the precise taxonomic identification of the rodent species and the associated hantavirus, which is required for the classification of viral species according to ICTV [19]. Nevertheless, the ongoing description of novel genera and species, especially cryptic species, has complicated taxonomic identification based only on morphological features; this issue has been resolved by using cytogenetic and molecular techniques [19,35,45,46].

Rodents are commonly classified based on cranial features, dentition and external morphology, including coat color and type, external metrics such as head-body, tail, ear (inner ear) and rear paw lengths, and data regarding body weight and age. This set of metrics varies between genera and species within a genus, making these measurements important for rodent identification [47,48].

Precise rodent identification has been made possible using cytogenetic and molecular taxonomic techniques. This advance occasionally makes taxonomic reclassification necessary due to the proposal of novel genera or species. Thus, over the last 10 years, the phylogenetic-based reorganization of rodents, as shown in several recent publications on the topic, has been conducted based on nuclear such as von Willebrand Factor (*vWF*), interphotoreceptor retinoid binding protein (*IRBP*), and mitochondrial (mtDNA) DNA markers such as cytochrome *b* (Cytb), NADH-dehydrogenase 4 (ND4), 16S and cytochrome c oxidase *I* (COI) [49,50,51,52,53,54,55,56].

The Cytb gene is the most commonly used DNA marker for resolving phylogenetic relationships and inferring boundaries between rodent species [56,57,58]. Mitochondrial and microsatellite DNA markers have been used in ecological studies to investigate modes of hantavirus transmission [59,60]. Markers such as these have demonstrated high resolution for detecting kinship within a small population of *bank voles* (vectors of the Puumala virus) and have demonstrated utility for screening maternal lineages [60,61].

The taxonomy of the Old World rodent fauna, including species from Europe, Indochina and West Africa, is well known based on studies using molecular markers. However, the diversity and complexity of rodent fauna, which is greatest among the New World rodents of the Americas, particularly the subfamily Sigmodontinae, has posed a challenge for researchers studying their taxonomy and phylogenetic relationships. The reclassification of existing taxa has occurred in genera such as *Oryzomys*, which was divided into ten genera [45]. Thus, despite requiring greater expense and additional laboratory work relative to traditional methods, a molecular-based approach of species identification is an efficient way to clarify taxonomic ambiguities. Taxonomic classifications are more robust when molecular techniques are used in addition to morphological classification [49,50].

#### 2.1.2. Sigmodontinae Subfamily

Taxonomic studies on sigmodontinae have led to ongoing descriptions of novel species [62]. Taxonomic revisions and large compilations include the restructuring of the systematic knowledge on this group [62,63,64,65,66,67,68].

Classically, the genera of the subfamily Sigmodontinae [69] were organized into distinct groups, most of which were formalized as tribes in zoological classifications. Since the 1990s, phylogenetic approaches have been widely used to define these groups [70,71,72,73,74,75] thereby shedding new light on their nature and limits [76].

Some rodents of the subfamily Sigmodontinae have generalist living habits; they are able to occupy a wide range of habitats, which makes them able to adapt to the conditions of rural regions and peridomestic areas enter dwellings, bringing these reservoirs into close contact with human populations [35,77]. These rodents are found in practically all habitats throughout the Americas and are distributed according to morphoclimatic domains and plant formations [78]. Although they often have nocturnal habits, some species are active during the day. They are classified based on their lifestyle as terrestrial, arboreal, fossorial or aquatic. In most Sigmodontinae species, the females can produce multiple litters per year, and reproduction can occur throughout the year in hot regions. Individuals typically live less than two years. The great reproductive potential of some species can result in robust population growth under certain conditions, followed by sudden population decreases due to the depletion of resources in a particular area or other factors such as increased parasitism or changes in reproductive patterns. Such fluctuations can be periodic and multiannual, depending on the ecosystem where the rodents live [37,77,79,80,81].

#### 2.1.3. Infection

Hantavirus infection in rodents is apparently subclinical and can lead to lifelong viral reservoir status [11,82]. Studies with natural reservoirs of various hantaviruses have confirmed the suspicion that infection can pass between rodents by horizontal transmission [48]. In addition to biting and aggression between individuals, aerosol transmission is also considered a significant mode of transmission between rodents [11,83,84,85,86,87]. Some viruses, such as the virus Sin Nombre, appear to be directly transmitted by bodily fluids, particularly during aggressive encounters and possibly during “allogrooming” (cleaning and licking between different individuals in a colony) [61,88]. However, many hantaviruses (PUU, SEO, BCC, HTN) are transmitted indirectly between hosts by inhalation of contaminated feces [84,89,90,91]. The lack of evidence for aggression in some *Oligoryzomys* species suggests that viral transmission can occur during communal nesting in the winter months [40,85]. Vertical transmission has not been demonstrated, and maternal antibodies (MatAbs) appear to protect offspring against infection for a few months. Infected females commonly transfer MatAbs to their offspring *in utero* and during suckling providing temporary protection that can last up to 80 days for PUUV in bank voles, which have an average lifespan of 3 to 5 months in the wild [92,93,94,95]. Therefore, the transfer of MatAbs can cause a delay in hantavirus transmission, given the large proportion of young individuals with temporary immunity, which can affect the infection dynamics for natural rodent populations [96].

Natural hantavirus transmission in rodents remains poorly understood. Although there is no evidence of vertical transmission, some studies have suggested the existence of more than one source of horizontal transmission, as described above, which may vary depending on the behavior of the natural hosts, the virus type and the ecosystem in which the rodents and virus circulating. In the natural environment, the seroprevalence among rodents typically increases with weight (relative age marker) and age, emphasizing the role of horizontal transmission in maintaining the virus within rodent populations [84,86,88]. Moreover, the high prevalence among males and the presence of injuries in some of the males highlight transmission via aggression and biting, which could occur during territorial disputes [35,85,86]. The effect of age, which is associated with a long exposure to the virus, supports the idea that horizontal transmission is the main mechanism for maintaining hantavirus in nature [97]. In short, sex, age, body mass, and wounds were important predictors regarding the probability of hantavirus infection within the rodent reservoirs populations [98,99].

Hantaviruses in rodent reservoirs are typically isolated from lung and kidney tissue. The viruses are concentrated and eliminated through saliva, urine and feces over a long period of time. The time during which the virus is eliminated in each type of excretion varies for each viral type and species of rodent infected [100,101,102,103]. During the experimental infection of the rodent *Sigmodon hispidus* with the Black Creek Canal (BCC) virus, the virus was detected in urine up to 70 days after infection [104]. For Puumala (PUU) virus in the rodent *Myodes glareolus*, the period of detection in the urine was 44 days, and the greatest levels of viral RNA were detected between 14 and 21 days after infection [105]. The authors detected the presence of viral RNA in saliva and feces up to 84 and 44 days after infection, respectively. Several studies have indicated that the main transmission route of hantavirus is aerosolized urine [106,107]. Recently, other studies have indicated the additional importance of saliva and feces for viral transmission [84,105,108]. The earliest report of virus detected in excretions was 5 days after infection in laboratory studies. Hantaviruses are generally most effectively eliminated during the first two months of the life of a rodent, but the elimination of virus in the wild can persist for up to 8 months, as shown in bank voles [94,109].

Hantaviruses cause persistent infections in rodents marked by a relatively short initial acute phase lasting two to three weeks, during which the concentration of infectious virus is high. Subsequently, the infection is marked by a prolonged chronic phase during which the infection is productive, but the virus is normally present at low levels that can vary cyclically, even in the presence of high levels of neutralizing antibodies [100,104,110,111,112].

Viremia is transitory in rodents, most likely due to the elimination of virus by the action of neutralizing antibodies that are not able to completely eradicate the infection [113,114]. Factors contributing to persistent hantavirus infections in rodents remain poorly defined [82,115]. In this sense, Schountz and colleagues introduced molecular techniques that allowed further investigations into the immunological mechanisms that lead to the persistence or clearance of hantavirus [114]. The detection of viral RNA at high levels in the blood of naturally infected rodents during the acute phase indicates that recently infected rodents play an important role in viral transmission within rodent populations in nature [103].

In addition to the lungs and kidneys, other organs such as the heart, spleen, liver and nervous system usually test positive for viral markers during the acute phase of infection. However, distinct patterns of infection that can be localized or distributed have been observed for different organs and even for the same virus [82,112]. Viral titre appears to vary depending on the host, organ and viral type according to Korva and colleagues [103]. The highest viral titres have been found in lung samples from rodents naturally infected by the Puumala and Dobrava viruses (~1.7 × 10^9^ copies/mL homogenised tissue) and in kidney samples from *Apodemus agrarius* rodents naturally infected with the Saraaema virus. The viral loads in the various tissues studied ranged from 10 to 10^11^ copies/mL. The range of the viral load in urine samples was 10 to 10^6^ copies/mL.

Although a lack of evidence for illness is considered a typical feature of hantavirus infection in rodent reservoirs, studies on wild rodents seropositive for the Sin Nombre and New York viruses showed a high prevalence of pulmonary edema and periportal hepatitis, which suggest the possibility that histopathological findings consistent with an intermediate form of HPS may occur in rodents infected in nature [100,110]. In addition to the evidence for histopathological changes, infection with the PUU virus has recently been shown to affect the survival of rodents during the winter months because the animal must allocate its limited resources to deal with the persistent infection [90,94,116] According to a demographic field data collected over 15 year, antibody-positive male deer mice to SNV are less likely to survive compared to uninfected mice [117].

Despite the lack of knowledge on the influence of antibodies on the establishment and maintenance of persistent asymptomatic infections in natural reservoirs, some studies have shown that rodents develop a strong immunological response to the N antigen, which contrasts the lack of antibody production against glycoprotein Gn (G1) [118,119,120]. Moreover, given that persistence of the virus depends on its ability to maintain itself and propagate by escaping the host immune response, the following mechanisms possibly related to viral refraction to the rodent immune response have been suggested: (i) the presence of a non-lytic infection; (ii) the ability of the virus to replicate in immune cells such as macrophages, monocytes and T lymphocytes; (iii) downregulation of the cyclic phases of viral replication; and (iv) the activation of regulatory T cells [82,121,122,123]. In addition to this anti-inflammatory profile of the immune response in rodent reservoirs involving the production of high titres of neutralizing antibodies, cytotoxic CD8^+^ T cells are absent in infected rodents, most likely due to immunosuppression, which is most evident in the lungs because the lungs represent the main site of viral replication. A similar scenario has not been observed in the spleen, an organ that conducts a strong inflammatory response, suggesting that the lungs exhibit less immunological activity during infection [113,121,122].

In some bank vole populations in Europe within regions where the virus is endemic, an inverse correlation is observed between the prevalence of PUUV infection and basal levels of pro-inflammatory tumor necrosis factor (TNF). In other words, the lack of an ostensive pro-inflammatory response in host reservoirs can play an important role in the persistence of hantaviruses in their host [114,124,125,126]. These studies suggest the existence of a subtle balance between an efficient immune response to limit viral replication and a strong uncontrollable response that results in fatal illness. Recently, an experimental Andes virus infection in deer mice, the reservoir of SNV, resulted in the viral clearance, by up-regulation of genes involved in the immune response, and by the production of antibodies. These findings support the hypothesis that hantavirus replication and persistence are restricted to the primary reservoirs in which they have adapted and that a persistence model of other host might lead to insights about what viral genetic changes are needed to develop reduce immunologic recognition of the virus and persistent infection [127].

#### 2.1.4. Geographic Distribution of Hantavirus Infections in Old World Rodent Hosts

Not all hantaviruses are found throughout the geographical distribution (host range) of their host species. This lack of congruence may be due to a combination of factors including the requirement of population density thresholds to sustain infection in the reservoir [128] or the existence of known cryptic host species that are unable to support infection by a specific virus [35,40]. Additional studies are required to understand the reasons for the apparent restriction of viral activity to a portion of the host range. The geographical distributions of viruses in their hosts are summarized in the tables shown in the present and following sections according to the taxonomic classification of the reservoirs.

In Asia and Europe the hantavirus are carried for two subfamilies of rodents, the Murinae (family Muridae) and Arvicolinae (Cricetidae). Rodents of the subfamily Murinae are found in Eurasia and Africa, except for the species *Rattus rattus*, *Rattus norvegicus* (Norway rat) and *Mus musculus*, which were spread across various continents after being introduced by European colonizers. In addition to the subfamily Arvicolinae, which is widely distributed across the Northern Hemisphere from Eurasia to North America, the murine rodents are the most important reservoirs of hantavirus that cause HFRS in the Old World.

The main reservoirs of hantavirus in the Old World include the following species: (1) rodents of the genus *Apodemus* associated with the hantaviruses Hantaan, Amur and Soochong in Asia and the hantaviruses Dobrava and Saaremaa in Europe [5,129,130,131,132]; (2) rodents of the genus *Rattus* in Asia (Seoul, Gou and Serang) [133,134]; (3) rodents of the genus *Bandicota* in Asia (Thailand) [135,136]; (4) hantaviruses associated with the genus *Myodes* in Europe (Puumala) [137]; and (5) rodents of the genus *Microtus* in Europe harboring the Tula virus [138,139,140,141] (see Supplementary Table 1).

The Norway rat is one of the most successful invasive vertebrates and inhabits urban and rural environments. A high density of *R. norvegicus* is commonly found in low-income neighborhoods within heavily populated cities [142,143]. The SEOV virus was detected among rats in Asia, Europe and the Americas [97,144,145] including Brazil [7]. The Serang virus is transmitted by the Asian house rat (*Rattus tanezumi*) [134] and is widely distributed in Southeast Asia.

Considering the discussions that have occurred over recent years due to a lack of agreement on the viral classification of different lineages, more sequences from various geographical lineages must be obtained and analyzed to clarify the genetic relationship between hantaviruses DOBV and SAAV. Originally identified in *A. agrarius* from the Baltic Islands, the hantavirus SAAV was subsequently found in the same reservoir from other areas of Europe (Central and Eastern Europe). Although typically considered a strain of SAAV, the DOBV virus is sometimes identified as DOBVAa. This fact, in addition to the recent discovery of *A. flavicollis* infected with the strain DOBV or DOBVAf in southeastern Europe (the Balkan region), suggests that further phylogenetic discussions on DOBV and SAAV are required [97]. Therefore, the ICTV approved SAAV and DOBV as viral species, and more recently, the hantaviruses DOBV and SAAV have been suggested to be subdivided into four related genotypes:Dobrava, Sochi, Kurkino and Saaremaa [146].

*Apodemus* rodents, which include *Apodemus flavicollis*, are widely present in most of Europe except for Scandinavia, the British Isles and Western Europe near the Atlantic Ocean. In the Far East and in China, *Apodemus agrarius koreae* (the Korean field mouse) is the vector for HTNV, which was originally discovered in 1976. *Apodemus peninsulae*harbors a hantavirus genetic variant related to HTNV called AMRV or SOOV [130,131,147].

The host for PUUV is the bank vole *Myodes glareolus* (previously *Clethrionomys glareolus*), which belongs to the family Cricetidae subfamily Arvicolinae [78]. The bank vole is one of the most abundant and broadly distributed mammalian species in Europe, imported from the British Isles through continental Europe and Russia to Lake Baikal. The range of the bank vole extends north to the Arctic Circle and south to parts of Northern Spain, Greece, Turkey and Kazakhstan. Although it is not found in the Southern Iberian Peninsula or Mediterranean islands, the bank vole is a widespread species found in European regions up to 2,400 m above sea level [148]. The bank vole inhabits a wide variety of forest habitats, including temperate broad-leaved and mixed forests, but it prefers areas with dense vegetation [149].

The genetic variability of PUUV features a geographical structure within the bank vole distribution [135,150,151,152,153,154,155,156,157,158,159]. Eight lineages of PUUV have been described in Eurasia: (1) the Central European (CE) lineage; (2) the Alpe-Adrian (ALAD) lineage; (3) the Latvian (LAT) lineage; (4) the Danish (DAN) lineage; (5) the South-Scandinavian (S-SCA) lineage; (6) the North-Scandinavian (N-SCA) lineage; (7) the Finland (FIN) lineage and (8) the Russian (RUS) lineage. The distribution of this chain of distinct PUUV lineages is thought to be based on the isolation of rodent populations during the last glacial maximum (LGM) and the subsequent recolonization of Eurasia [158].

The Tula virus (TULV) is widespread in Eurasia, including France, Germany, Netherlands, Austria, Slovenia, Croatia, Hungary, Poland and Russia, where numerous mammalian species, including *M. arvalis*, *M. subterraneus*, *M. rossiaemeridionalis*, *M. agrestis*, *M. gregalis*, *A. amphibius* and *Lagurus lagurus*, have been identified as reservoirs [140,160]. In the Balkans, Serbia was the first country in which TULV was detected in the European pine vole *M. subterraneus* (*Pitymys subterraneus*) [161].

In addition to the host genera mentioned above, hantaviruses have been found in *Hylomyscus* and *Niviventer* (Murinae) and *Eothenomys* and *Lemmus* (Arvicoline). Furthermore, evidence suggests that the transfer of hantavirus from one species to another occurred during the evolution of hantaviruses in the Old World (for example, between *Microtus* carrying VLA or KHAV and *Lemmus* carrying the Topografov virus [TOPV] [43,139].

#### 2.1.5. Geographic Distribution of Hantavirus Infection in New World Rodent Hosts

**Figure 1:**
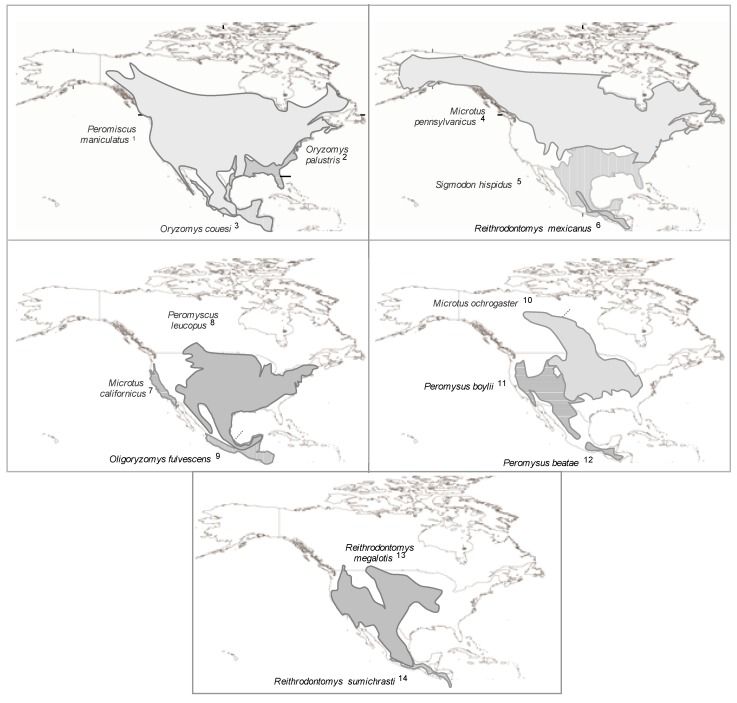
Distribution of the North American hantavirus primary reservoirs. Number after species names refer to the hantavirus genotype or specie (*) associate: (1) Sin Nombre virus* and Monongahela virus; (2) Bayou virus*; (3) Catacamas virus and Playa de Oro virus; (4) Prospect Hill virus; (5) Black Creek virus* and Muleshoe virus*; (6) Rio Segundo virus*; (7) Isla Vista virus; (8) Blue River virus and New York virus*; (9) Choclo virus; (10) Bloodland Lake virus; (11) Limestone canyon virus; (12) Montano virus; (13) El Moro Canyon virus* and Huitzilac virus; (14) Carrizal virus, see Supplementary Table 2.

**Figure 2:**
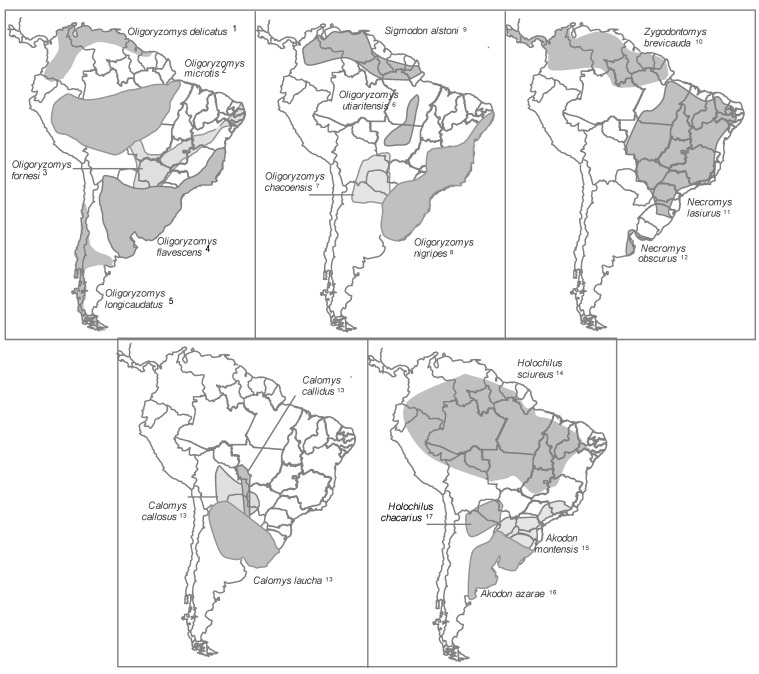
Distribution of the main South American hantavirus reservoirs. Number after species names refer to the hantavirus genotype or specie (*) associate: (1) Maporal virus; (2) Rio Mamore virus*; (3) Anajatuba virus; (4) Central Plata virus and Lechiguanas virus; (5) Andes virus* andOran virus; (6) Castelo dos Sonhos virus; (7) Bermejo virus; (8) Juquitiba virus and Itapua virus; (9) Caño Delgadito virus*; (10) Calabazo virus; (11) Araraquara virus; (12) Maciel virus; (13) Laguna Negra virus*; (14) Rio Mearin virus; (15) Ape Aime-Itapua virus and Jabora virus; (16) Pergamino virus; (17) Alto Paraguay virus, see Supplementary Table 2.

All hantaviruses that cause HPS in the Americas are linked to rodents of the subfamilies Sigmodontinae and Neotominae, which are endemic to the Americas [77,162,163]. Most rodent genera associated with hantaviruses transmitted by Sigmodontines are endemic to South America, with some exceptions for parts of North America. The hantaviruses transmitted by Neotomine are found in *Peromyscus* and *Reithrodontomys*, and both genera are endemic to Central and North America (Figure 1 and Figure 2).

In the New World, more than 30 new genotypes have been described, and 11 are officially recognized as viral species by the ICTV [164]. The main reservoirs include the following:

(1) rodents of the genus *Peromyscus* in the USA and Canada, associated with the hantaviruses Sin Nombre, New York, Monongahela and Montano. The Montano virus is found in Mexico [165,166,167]
(2) rodents of the genus *Oligoryzomys*, associated with the hantaviruses Andes (ANDV), Oran, Lechiguanas and Bermejo in Argentina; ANDV in Chile; Choclo in Panama; Central Plata in Uruguay; and the genotypes Juquitiba, Castelo dos Sonhos, Anajatuba and Rio Mamore in Brazil [18,168,169,170,171,172]
(3) rodents of the genus *Calomys*, which are reservoirs for Laguna Negra in Paraguay, Bolivia and Brazil [173,174];
(4) rodents of the genus *Necromys*, which are reservoirs for the hantaviruses Maciel in Argentina and Araraquara in Brazil [169,170];
(5) rodents of the genus *Akodon*, which are reservoirs of the viruses Pergamino in Argentina and Jabora in Brazil and Paraguay [169,175,176,177]
(6) rodents of the genus *Holochilus*, which are reservoirs of the viruses Rio Mearim in Brazil and Alto Paraguay in Paraguay (See Supplementary Table 2).

Many hantaviruses have been identified in more than one potential host, and additional studies are required to clarify the roles of host specificity, spillover, host-jumping and cospeciation with respect to hantavirus-host association. The Laguna Negra virus appears to be associated with the three related host species *Calomys laucha* in Chaco Paraguay [178], *Calomys callosus* in Northern Argentina [179] and Bolivia [180] and *Calomys callidus* in Central-Eastern Brazil [174]. Similarly, after the first description of the Limestone Canyon virus in *Peromyscus boylii*, discovered in northern Arizona, USA [181], the same genotype was detected in *P. hylocetes* and *Peromyscus melanotis* in Mexico, *P. levipes* in Nuevo León, *P. ochraventer* in San Luis Potosí, and *P. spicilegus* in Jalisco [182]. Although these are natural hosts, these species are not necessarily the main host for the virus.

Hantaviruses are common in South America and are often related based on their amino acid sequence. The patterns of diversity for hantaviruses in South America are more complex relative to viruses described in North America, which is likely due to recent and rapid evolution among South American rodents and the complexity phylogenetic and taxonomic relationships between southern sigmodontinae species and the hantaviruses associated [40,183,184]. Many discrepancies exist between the phylogenies of the S and M segments for South American hantaviruses, which suggests the occurrence of many rearrangement and host-switching events [185]. In agreement with *in vitro* rearrangement studies on the Andes virus, according to Chu *et al*., the Ape Aime genotype identified in *Akodon montensis* originated from a rearrangement of the S segment from the Jabora viral genotype (*A. montensis*) and the M segment from the Pergamino viral genotype associated with *Akodon* azarae [186]. *Akodon* rodents inhabit pastures in the Pampas and Atlantic forests in Argentina, Uruguay, Southern Brazil, Bolivia and Paraguay [179,187]. Given the complex and still relatively poorly understood systems of many rodents, especially in South America, in addition to the reports of small samples for some taxa, publishing accurate information on their geographical distributions remains difficult. Nevertheless, various species that are thought to be distributed across large geographic areas (for example, *O. longicaudatus* and *O. flavescens*) are being recognized as species complexes or subspecies [40,188,189,190]. Moreover, other broadly distributed species (for example, *C. laucha*) are being carefully studied taxonomically. Thus, the known distributions of primary rodent hosts were outlined (Figure 1 and Figure 2) to attempt to describe the potential area for each viral genotype known in the Americas based on the assumption that the distribution of each virus coincides with the distribution of its rodent host [35]. Nevertheless, several hantaviruses does not occur throughout the currently recognized range of its hosts [40].

In North America, the Bayou virus appears to occur throughout most of the geographical distribution of its primary host, *Oryzomys palustris* [191]. In contrast, the Black Creek Canal virus has been found in a limited area of Southern Florida although the host rodent species, *Sigmodon hispidus*, is found throughout the southeastern USA [119].

Mills (2006) have suggested that the host species of zoonotic viruses such as hantaviruses are more likely to be opportunistic and generalistic species that often inhabit anthropogenically altered environments with a clear gradient of tolerance to the level of habitat disturbance, and there seems to be a gradient tolerance for this change (habitat disturbance) [33]. For example, the rodent species *C. laucha* appears to occur only in disturbed crop-field habitats. Moreover, the prevalence of infection in some host species appears to vary across habitats, as found in the species *O. flavescens* in Uruguay, where seropositivity is more prevalent in environments that have been modified by humans (agricultural ecosystems, roads, shrublands, artificial woodlands and the areas around dwellings) than in undisturbed habitats [192]. In Panama, specific crop microhabitats near human habitation provided high-risk sites for human infection because of the elevated relative abundance and numbers of seropositive *O. fulvescens* [193].

##### 2.1.5.1. Rodent Reservoirs in Brazil

In Brazil, new species of the order Rodentia have been recognized, described and even reclassified. Some 71 genera and 235 species of rodents have been found in Brazil. The family Cricetidae is the most diverse, with 117 species and 36 genera grouped in the subfamily Sigmodontinae, which includes all hantavirus hosts identified in Brazil [48,194].

This broad diversity of Sigmodontinae is found in the Amazon, Cerrado (a savannah-like ecosystem), Atlantic Forest, Caatinga, Pampa (Southern Plains) and Pantanal formations. In these different types of regions, one can find widespread and restricted rodent species, as well as generalist and specialist species, with respect to habitat use [195,196]. Notably, in most of Brazil, all the aforementioned biomes have experienced rapid fragmentation, which may affect the ability of rodent species to spread throughout the landscape and may even affect species substitution [197,198]. This situation threatens the great diversity and many endemisms of these biomes thereby affecting hantavirus transmission dynamics.

Anthropogenic habitats are colonized almost exclusively by rodent species with generalist habits and often by non-autochthonous species, both can reach high population densities and are frequently competent hantavirus reservoirs [199].

**Figure 3:**
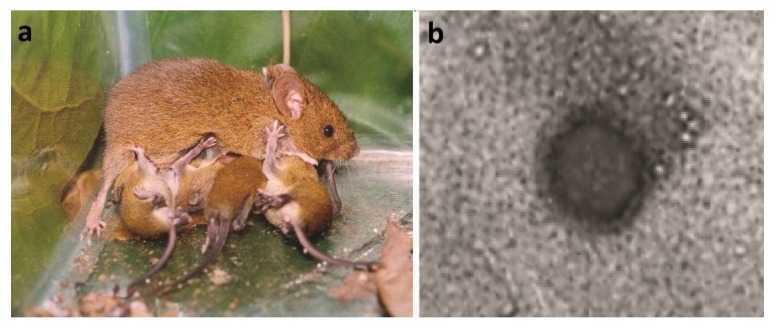
(**a**) *Akodon montensis*. Image courtesy of Cibele Bonvicino, Ph.D. (**b**) Electron micrograph. Morphology of Jabora hantavirus particule obtained from lung sample of *Akodon montensis*. Scale bar: 100 nm. Image courtesy of Debora Barreto, Ph.D. and Monika Barth, Ph.D.

In Brazil, knowledge regarding HPS has advanced greatly in recent years in terms of clinical and epidemiological findings related to the disease and in terms of the identification of novel viruses and their host species through cross-sectional studies and more recent studies on the ecology of reservoir populations. In addition to the hantaviruses that cause HPS (Juquitiba and Araraquara), which were previously characterized and related to the rodents *Oligoryzomys nigripes* and *Necromys lasiurus*, respectively, three novel hantavirus genotypes have been identified: the Anajatuba, Castelo dos Sonhos and Laguna Negra-like viruses associated with the rodent species *Oligoryzomys fornesi*, *Oligoryzomys utiaritensis* and *Calomys callidus*, respectively [171,174,200,201]. Subsequent genetic analyses of samples from rodents captured at sites of human infection broadened the genetic description of Juquitiba*-*like viruses with a description of the Araucaria variant in the rodent *O. nigripes* in Paraná state, where co-circulation of this viral lineage was demonstrated in the species *Oxymycterus judex* and *Akodon montensis* [38]. These genetic analyses also identified novel hantaviruses considered non-pathogenic in various wild rodent species, including the Rio Mearim virus in the rodent *Holochilus sciureus*, the Jabora virus in *Akodon montensis* (Figure 3) and the Rio Mamore virus in *Oligoryzomys microtis* [18,171,177].Among all hantaviruses genotypes described in Brazil, only Laguna Negra virus and Rio Mamore virus are currently recognized as species by ICTV (Supplementary Table 2).

Some features of habits and geographical distributions that are considered important are briefly discussed below to improve our understanding on the role of rodent reservoirs.

**Figure 4:**
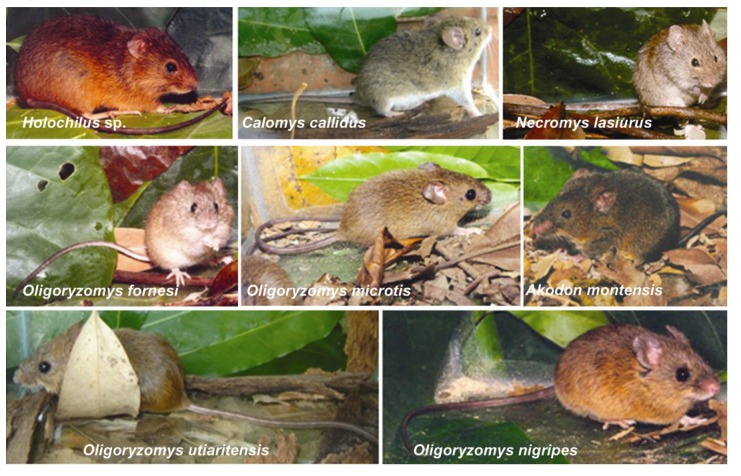
Rodents reservoirs of the hantaviruses described in Brazil. Image courtesy of Cibele Bonvicino, Ph.D.

***Oligoryzomys nigripes*** (black-footed pygmy rice rat)—this species is a reservoir for the Juquitiba virus. *O. nigripes* is a rodent with a generalist habit that inhabits forests and open vegetation formations and has a great capacity for adaptation to anthropic environments, including agricultural areas (especially corn plantations), where the species is most abundant [202,203]. These rodents can easily invade dwellings and barns. This species is abundant in the Atlantic Forest and Araucaria forest areas throughout Southern Brazil and the Southern part of the gallery forests of the Cerrado, extending to the Pampas on the southern edge of Brazil (Southern Plains) [203,204]. *O. nigripes* has been isolated at elevations ranging from 100 m [205] to 2000 m [206] in the Atlantic Forest (Figure 4).

***Oligoryzomys fornesi***—this species harbors the Anajatuba hantavirus and features a broad geographical distribution but mainly occurs in the Cerrado, the Caatinga, at the edge of the Amazon biome, and in altered or conserved vegetation, where it is common but not abundant. *O. fornesi*may be syntopic with *O. nigripes* [205] (Figure 4).

***Oligoryzomys utiaritensis***—this rodent harbors the Castelo dos Sonhos hantavirus and inhabits the Southern part of the Amazon biome and the Cerrado transition zone [49] (Figure 4).

***Oligoryzomys microtis***—this species harbors the Rio Mamore virus and occurs across nearly all of North Brazil especially South of the Solimões-Amazon rivers. *O. microtis* may be found in much of the Amazon biome on the edge of riparian forests [48,78,207] (Figure 4).

***Necromys lasiurus*** (hairy-tailed bolo mouse)—this species harbors the Araraquara virus. Its geographical distribution in Brazil includes the Cerrado, the Atlantic Forest-Cerrado ecotone and Caatinga areas. *N. lasiurus* can adapt to anthropic environments, especially grasses (*Brachiaria*) and sugarcane crops. These rodents are generally abundant but do not colonies human dwellings; however, they may occasionally invade homes [48,78,208] (Figure 4).

***Holochilus sciureus*** (Amazonian marsh rat)—this rodent is the host for the Rio Mearim virus and has a semi-aquatic habit feeding mainly on riparian grasses and sugarcane leaves. *H. sciureus* inhabits open areas such as fields, swamps, forest glades and agricultural lands at elevations up to 2000 m (6600 feet). *H. sciureus* has a broad geographic distribution and can be found in the Amazon, Caatinga, Pantanal and Cerrado biomes [48,78] (Figure 4).

***Akodon montensis*** (Montane grass mouse)—this species harbors the Jabora hantavirus(Figure 3 and Figure 4) and can be common or abundant in forests and cultivated land in southern and southeastern Brazil throughout the Atlantic Forest and the Southern Plains (Pampas). This species generally lives in burrows composed almost entirely of layers of decomposing leaves (litterfall), in which they dig tunnels. At higher elevations (above 900 m) *A. montensis* lives under grasses and maintains an insectivorous-omnivorous diet [78,209,210,211].

***Calomys callidus***—this rodent harbors the Laguna Negra-like hantavirus and inhabits Cerrado transition and Amazon areas in the States of Mato Grosso and Rondônia [47,174]. This region has suffered intense deforestation for the planting of soy and other cereals (Figure 4).

Considering the continental extent of Brazil and the high biodiversity of sigmodontine rodent species, novel hosts and viruses with the potential to cause human disease could be identified as new studies and appropriate eco-epidemiological monitoring are conducted.

In addition to the hantaviruses associated with sigmodontines, ten years before HPS was recognized in 1983, the first hantavirus in Brazil antigenically associated with the Seoul virus was identified from the viscera of a rodent of the species *R. novergicus* [7]. Although the Seoul hantavirus has been identified in murine populations of the species *R. rattus* and *R. novergicus* in port cities around the world, the presence of HFRS had never been proven in Brazil, where *R. rattus* and *R. novergicus* are non-native, despite serologically positive human and rodent samples.

### 2.2. New Reservoirs

#### 2.2.1. Insectivores

More than 40 years after the identification of the Thottapalayam (TPM) virus, which was isolated in 1964, novel hantaviruses in other shrew and mole species have been identified in Eurasia, Africa and the USA [20,21,22,23,24,25,26,28,212]. To date, these hantaviruses have not been linked to HPS or HFRS, although antibodies against the TPM virus were observed in a feverish patient in Thailand [213].

Because of their small body size and because they share ecological communities, shrews and moles are occasionally confused with rodents despite having independent evolutionary histories [214].

In the order Soricomorpha previously called Insectivora, the shrews and moles belong to four families: Soricidae (shrews), Talpidae (moles), Solenodontidae (solenodons) and Nesophontidae (Nesophontes) [215]. To date, 21 hantaviruses have been described in five genera of shrews from the family Soricidae (*Neomys*, *Suncus*, *Crocidura*, *Sorex* and *Anourosorex*) and four genera of moles and shrew-moles from the family Talpidae (*Talpa*, *Urotrichus*, *Neurotrichus* and *Scalopus*) (see Supplementary Table 3).

Shrews are distributed globally except for New Guinea, Australia and New Zealand, where there are no native shrews. Shrews have been relatively recently introduced to South America and are present only in the Northern Andes [215]. In terms of species diversity, the family Soricidae is the fourth most successful mammalian family behind only the rodent families Muridae and Cricetidae and the bat family Vespertilionidae.

Shrews are generally terrestrial, feature a high metabolic rate and feed on seeds, insects, fruit pods, worms and a variety of other foods among the litterfall and dense vegetation [216]. Some species of shrew are specialized for climbing trees, whereas other species live underground or under the snow [217]. Shrews do not hibernate but can enter torpor. Therefore, many species of shrew dramatically decrease in body size during the winter [218]. Shrews which only meet to breed can live from 12 to 30 months [217].

Moles and shrew-moles (Talpidae) occur throughout the Northern Hemisphere, South Asia, Europe and North America except for Mexico and Ireland [215]. Moles appear to be misanthrope animals, although at least one species, the star-nosed mole, shares burrows.

Shrew-moles share physical features with shrews, such as small body size, paired ampullary glands, posteriorly directed pelage and a long pointy nose. They also share physical features with, moles such as strongly insectivorous dentition, large head and enlarged forefeet [219]. Shrew-moles are active throughout the day and night (generally travelling above ground only at night) with intermittent periods of rest and/or sleep [220]. They build networks of shallow and deep underground burrows and tend to be social, unlike most solitary and reclusive shrew species.

Four hantaviruses have been identified in shrew-moles: the Asama virus in *Urotrichus talpoides*, which occurs only on Japanese plains and islands; the Oxbow virus in *Neurotrichus gibbsii*, which is the smallest and most primitive mole in North America and can also be found in the western parts of Canada and the USA up to central California, where the Oxbow virus is sympatric and syntopic with other shrew and rodent hantavirus hosts such as the deer mouse; and the Cao Bang and Lianghe viruses, both of which have been identified in the Chinese shrew mole *Anourosorex squamipes*, which is an animal that inhabits forests located between 1500 and 3000 m in elevation and has a vast geographic range extending from central-western China to northern Myanmar, northern Thailand, Assam, Bhutan, North Vietnam, Taiwan and possibly Laos [24,29].

Rockport virus (RKPV), a hantavirus identified in archival tissues of the fossorial eastern mole (*Scalopus aquaticus*) in 1986, shared a most recent common ancestor with cricetid-rodent-borne hantaviruses, raising the possibility of host-switching events in the distant past. Although Eastern moles are generally solitary they may share burrows or tunnels with other moles in areas where their home ranges overlap [26,27,29].However, their low population density and underground lifestyle relative to the high population density and above ground lifestyle of sympatric rodent species generally offer limited opportunities for direct contact. Nevertheless, hantavirus transmission across species can occur by contact with contaminated secretions and excreta. Detection of the Nova virus in renal tissue of the European mole (*Talpa europaea*) suggests the possibility of viruria [197]. Within this context, Imjin virus (MJNV) infection among adult males of the shrew species *Crocidura lasiurus* in South Korea is generally overrepresentation; however, whether this predominance reflects intraspecific transmission through aggressive encounters (injuries) remains unknown [221].

Some shrew species display aggressive behavior and have carnivorous habits, such as the species *Sorex araneus*, which harbors the Seewis hantavirus. These features increase the possibility of acquiring and transmitting hantavirus infections through injuries. Additionally, shrew species are found in forests, fields and hedgelands throughout Northern Europe including Scandinavia and Great Britain and extending throughout Russia. Thus, the shrew species that act as hantavirus reservoirs increase the possibility of host-switching events [23], as shrews are recognized as one most widespread species of small mammals in Eurasia. Shrews occasionally occupy the burrows of rats, voles and moles, increasing the potential for host-switching.

Due to the sympatric and synchronic coexistence of some shrews, such as the species *Blarina brevicauda*, which inhabits the mid-eastern USA and harbors the Camp Ripley virus with Neotominae and Arvicolinae in North America, the possibility of infection by spillover cannot be ruled out [25]. Another example is the dusky shrew (*Sorex monticolus*). This shrew specie harbors the Jemez Springs virus (JMSV), which was identified in four soricidae species, including the masked shrew (*Sorex cinireus*), which has also been found to harbor the Ash River virus (ARRV) in the USA [222]. The masked shrew and dusky shrew are the most widespread shrews in North America, and they occupy a variety of humid habitats. Therefore, the sharing of nesting materials and inter- and intra-specific disputes can result in infection by spillover. Notably, a virus very similar to JMSV was identified in another shrew species *S. haydeni* in New Mexico [222]. Additionally, viruses antigenically related to the Hantaan virus have been isolated from *Suncus murinus*, *Crocidura russula* and *Anourosorex squamipes*, which suggests that shrews can act as accidental hosts for hantaviruses typically found in rodents [25,223,224].

The genus *Crocidura* (Soricidae) is widespread throughout Europe, Africa and Asia and contains more species described than any other mammalian genus. The species that comprise this genus include the following: (i) *C. lasiura*, which is associated with the MJNV virus in Korea and can be found in forest glades, wetlands, dry meadows, shrub forests, along the banks of rivers and lakes, in agricultural fields and on roadsides [221]; (ii) *C. theresae*, which is associated with the Tanganya virus (TGNV) in Guinea and inhabits forests of tropical or subtropical humid lowlands and humid savannahs [22]; (iii) *C. obscurior*, which is a West African pygmy shrew found in humid subtropical and tropical regions with low altitude forests in Ghana, Guinea, Liberia, Sierra Leone and possibly Nigeria and is associated with the Azagny virus (AZGV) on the Ivory Coast [29]; and (iv) *C. shantungensis*, which is a species associated with the Jeju virus (JEJV) on Jeju island off the southern coast of Korea [225].

The recent demonstration that TPMV and other hantaviruses identified in Soricidae and Talpidae are genetically distinct, combined with the detection of virus in tissues from these animals originating from very distant geographical regions, challenges the conventional view that rodents are the primary or primordial host reservoirs for hantavirus. Furthermore, this evidence suggests an ancient coevolution and adaptive radiation within a complex evolutionary scenario where host-jumping and co-divergence appear to have played a critical role [19,29,222]. Within the framework of molecular phylogeny and zoogeography, the tight association between distinct hantavirus clades and specific subfamilies of rodents, shrews and moles appears to have resulted from alternating and periodic codivergence over evolutionary time.

#### 2.2.2. Bats

Recent studies have demonstrated the potential for bats to act as hantavirus hosts. After the initial identification of a novel hantavirus called the Magboi virus in bats of the species *Nycteris híspida* (slit-faced bat) in Sierra Leone [31], studies identified the presence of another hantavirus called *Mouyassué* in bats of the species *Neoromicia nanus* (banana pipistrelles) in Africa [30]. More recently, three novel hantaviruses were identified: the Longquan virus in *Rhinolophus* bats of the species *R. affinis*, *R. sinicus* and *R. monoceros* and the Huangpi virus in *Pipistrellus abramus* in China and Xuan Son virus in *Hipposideros pomona* in Vietnam. These findings demonstrate the potential, diversification and need for further studies on these natural hantavirus hosts [226,227].

Bats (order Chiroptera) are sources of a broad range of emerging pathogens, including coronavirus, filovirus, Hendra and Nipah paramyxoviruses and lyssavirus [228]. With fossil origins from the Eocene epoch approximately 50 million years ago, bats occur on all continents except Antarctica and are one of the most species-rich mammalian orders, totaling over 1100 [229].

The characteristics of bats, such as global distribution, abundance, flight ability (some species can cover more than 80 km per night while foraging), high population densities and sociability in some species, favor the maintenance, evolution and spread of viruses. Many bat species are gregarious and form groups of a few individuals or colonies that can reach up to 20 million individuals, such as the species *Tadaria brasiliensis* in Bracken Cave in Texas, USA [230]. Additionally, different bat species can cohabitate in the same shelter, allowing the possibility for interspecific transmission and a high rate of contact within these colonies that can lead to rapid transmission of pathogens.

Other characteristics that make bats important reservoirs include their relatively long lifespans relative to body size, which can facilitate viral persistence and transmissibility, and their ability to hibernate or enter a state where their metabolism slows and they enter a prolonged torpor that leads to reduced viral replication and immune function [231,232].

Many bat species have peri-domiciliary habits and use human buildings as shelters by perching in houses, buildings or even cavities in the trunks and branches of trees within urban areas, leading to frequent human contact with bat droppings [233,234]. More studies, such as that conducted by Luis *et al*. [232] to compare the abilities of bats and rodents to function as viral reservoirs, are needed to clarify the potential importance of bats as hantavirus hosts. Notably, human contact with bats has increased in recent decades due to habitat invasion and greater use of bats as bushmeat [235,236,237].

Interestingly, all novel hantaviruses have been identified in insectivorous bat species. Unlike frugivorous, nectarivorous, carnivorous and hematophagous species, these bats are found in tropical and subtropical climates in nearly all regions of the globe, making insectivorous bats the targets of new discoveries in the hantavirus field [30,31,226,230].

The insectivorous bat species *Neoromicia nanus* (virus *Mouyassué*), which is widespread in the forests and savannahs of sub-Saharan Africa, is one of 13 species in the genus *Neoromicia* subfamily Vespertilioninae (Vespertilionidae). Unlike some frugivorous species, the banana pipistrelle is not traditionally eaten in Africa but can be found living within or near human dwellings, facilitating occasional human exposure to infected bats [30].

The vesper bat *Pipistrellus abramus* has been shown to harbor the Huangpi virus in China and occurs in Japan, Korea, Taiwan, Vietnam, Laos, Myanmar and possibly Russia. *Nycteris hispida* (carrying the *Magboi* virus) is an insectivorous bat of the family Nycteridae that is very common and inhabits a wide range of habitats, such as humid tropical forests, humid and dry savannahs and wetlands throughout much of eastern, central and western Africa. This species forms small colonies perched in hollow trees in caves and buildings [229].

Three bat species of the family Rhinolophidae have been shown to harbor the Longquan virus: *Rhinolophus sinicus*, which is known as the Chinese Rufous Horseshoe Bat and is widespread across China, India, Nepal and Vietnam; *Rhinolophus affinis*, which is known as the Intermediate Horseshoe bat and is distributed throughout much of South Asia; and *Rhinolophus monoceros*, which is endemic to Taiwan and China [226]. Previous studies collectively suggest that bats can act as natural hantavirus reservoirs and are therefore a potential wild mammalian reservoir for hantaviruses that could be associated with human disease.

Although confirmation is required, based on extensive sampling of mammalian taxa, previous studies have suggested that hantaviruses appeared in bats before colonizing rodents [226]. Evidence supporting this idea includes the fact that bats are evolutionarily older mammals than rodents and that the viruses that coevolved with bats could have theoretically used highly conserved cell receptors to increase their ability to colonize other mammals [228].

The hantaviruses identified in bats differ from other known hantaviruses, suggesting that bats are a natural host for the viruses, although interspecific transmission (spillover) from another host cannot be ruled out. Interspecific transmission was confirmed based on the detection of the Hantaan virus in bats from Korea and more recently in Brazil, which suggests the necessity of reproducibility of this observation [238,239]. In Brazil, there are only two studies that identified hantaviruses in non-rodents reservoirs: bats (*Diphylla ecaudata* and *Anoura caudifer*) and marsupials (*Micoureus paraguayanus*, *Monodelphis iheringi* and *Didelphis aurita*). Sequencing the S segment of the hantavirus from both animals revealed 95% identity with the Araraquara virus, initially described in *N. lasiurus* rodent [238,240]. The coexistence of these animals in the same area reinforces the possibility of interspecific transmission mentioned above.

Brazil is home to approximately 174 microchiropteran species (nearly 14% of the total species), a majority of which are insectivorous (112 species) [241], including bats of the family Verspertilionidae; thus the identification of novel hantaviruses in new reservoir bat species among the several Brazilian biomes remains a distinct possibility.

## 3. Risk Factors for Hantavirus Transmission to Humans

Hantavirus infections have most commonly affected low-income people in rural areas, especially males, because this demographic is associated with precarious living conditions and agricultural and/or reforestation activities, favoring frequent contact between humans and rodents [13,163,242,243].

Hantavirus infection has also been observed in peri-urban areas (periphery of villages and towns) with large populations and low sanitary standards, at least in part because they are infested with rodents that share space and food sources with the human populations [242,244]. Growing urbanization of areas that are considered rural, camping in the wilderness and other outdoor recreational activities (ecotourism and fishing) and the activities of professionals such as veterinarians, biologists, engineers and agronomists, among others, have facilitated the transmission of hantavirus to middle- and high-income persons [245,246,247,248].

In the Old and New World, outbreaks of hantavirus infections have been mainly associated with changes in rodent population density, which can vary significantly over time (seasonally or annually, depending on external factors such as interspecific competition, climate changes and predatory anthropic activities). These factors, which affect rodent population sizes and age structures and can directly or indirectly regulate viral prevalence and transmission in host reservoirs [35,249] were classified by [250] into the following five classes:

(1) environmental regulators (climate and resource availability) that modulate transmission rates through effects on reproductive success and population density;
(2) anthropogenic factors such as environmental disruptions that affect ecosystem complexity and favor opportunist or generalist species that can act as hantavirus reservoirs;
(3) genetic factors that can affect transmission;
(4) behavioral factors such as intra- or inter-specific aggression;
(5) physiological factors that control the response to and duration of infection in the host.

These five factors are important in the biology and ecology of the virus-host ecosystem and may ultimately reflect the risk of human transmission.

The changes that affect rodent population dynamics are often related to climatic effects. The best example was the Four Corners epidemic of 1993 in the Southwestern USA, which was preceded by a considerable increase in rainfall associated with the El Niño phenomenon in 1992 and 1993. The abundance of rain led to greater food supply for rodents and a consequent 20-fold increase in the size of the local rodent population, which resulted in the invasion of human dwellings by rodents and a significant increase in the risk of human infection [249,251,252].

Interestingly, global warming appears to have had opposing effects on the most common hantavirus PUUV and its host *Myodes glareolus* in various geographical regions across Europe. In central and western Europe, the population density peaks for bank voles are related to “mast years” or years when large quantities of seeds are produced by various species of European tree species (*Quercus petraca*, *Carpinus betulus* and *Fagus sylvatica*). These seeds accumulate in the soil, thereby increasing the food supply for rodents.

Recent studies conducted in Belgium demonstrated that high temperatures in the summer and fall for two years and one year, respectively, before the occurrence of illness (NE) led to a greater incidence of human cases of hantavirus. This correlation could be explained by the fact that hotter summers cause pine tree flowering. Combined with higher seed production and milder temperatures in the fall, this flowering led to greater survival of rodents through the winter. The arrival of the breeding season during the following spring with a high population density could explain the NE outbreaks associated with the PUU virus in the region. Years with greater incidence of NE exhibited a periodicity of two years in Belgium and other central European countries [253,254,255]. In contrast, milder winters in Scandinavia led to a decrease in the populations of rodent reservoirs as a result of little protective snow cover, combined with thawing periods that produced ice instead of snow cover [256,257].

However, hantavirus epidemics in western Europe cannot be exclusively related to “mast years” for a particular tree species due to the greatly differing number of cases recorded in recent years in neighboring countries, where these seed abundance events occur simultaneously [81].

A study conducted in Russia on wild rodents of the genus *Apodemus* revealed greater viral replication in summer and spring months, when the rodents are most active, which increases the transmissibility index [258]. A similar experimental study in Chile on rodents of the species *Oligoryzomys longicaudatus* in captivity showed that more horizontal transmissions occur during summer and spring [84].

Similarly, studies conducted in China demonstrated that good seed harvests, low rainfall rates or the lack of flooding and an abundance of the striped field mouse (*Apodemusagrarius*) as a result of these factors contribute to a greater incidence of HFRS cases [259,260].

Outbreaks related to urban rats of the genus *Rattus* are linked to various risk factors such as precarious living conditions and other behavioral factors for human populations, combined with a high density of rodents. HFRS cases generally occur throughout the year, with a greater prevalence in the Winter associated primarily with the HTN virus transmitted by field rodents and a greater prevalence in the Spring associated with the SEO virus carried by urban rats (*Rattus* spp.) [244,261,262].

Recent studies have suggested that the landscape composition is an important factor in the ecology of the hantavirus-host ecosystem. Habitat structure can also affect host reservoir population density by regulating the availability of cover or den space and other habitat elements that tend to impact the survival and proliferation of the host species. The large continuous forests of northern Europe can favor the efficient propagation of viruses through reservoir populations relative to the more fragmented forests of central Europe. Studies demonstrated a positive correlation between anthropic disturbances in the ground cover and the presence of hantavirus in a variety of ecosystems [32,263]. Goodin *et al*. investigated features of the habitat for the natural hantavirus host rodent *Akodon montensis* in Paraguay and found that *A. montensis* prefers forested areas with a small canopy and dense ground cover. The authors also found a significant difference in the microhabitats occupied by rodents, depending on whether they were seropositive or seronegative for hantavirus, indicating that habitats with greater overstory cover may promote transmission and maintenance of hantavirus in *A. montensis* [187].

In addition to climate, indirect effects on rodent population dynamics that promote greater risk of human exposure to hantavirus can be attributed to changes in human behavior, such as alterations due to the occupation of new environments, deforestation or reforestation, agriculture, civil engineering and other human activities that alter the spatial and temporal dynamics of the virus-reservoir ecosystem.

Local epidemics occur in foci or regions where the climate, biotic and abiotic conditions promote an increase in reservoir species and their contact with humans. Thus, long-term ecological studies that assess the prevalence of hantavirus in rodent hosts are essential for understanding hantavirus dynamic transmission with respect to the environmental (climate conditions and landscape composition) and ecological (virus-reservoir interactions) contexts and with respect to human risk behavior that differ between the various regions in which illness is reported.

The emergence of outbreaks or isolated cases of illness in Brazil have been linked to particular situations and environmental risk factors [200,264,265]:

(1) agriculture profile, as found in most cases in the southern states that involve corn fields that border gallery forests;
(2) an association with the construction of stockpiles or other attachments that permit the entry of rodents and consequently direct access to the stored food or grain;
(3) managing corn fields when using “direct seeding” and when keeping part of the harvest (cobs or bags of shelled corn) at the planting site, thereby allowing access by wild rodents that leave their droppings in the corn;
(4) significant ecological imbalances such as deforestation combined with the near extinction of natural predators for rodents (snakes, hawks, owls, *etc*.), leading to a population increase and subsequent invasion of dwellings and attachments in rural areas when the food supply becomes exhausted;
(5) areas reforested with pine and eucalyptus, where the wild rodent population is adapted to the new habitat, consequently increasing contact with humans during the extraction of timber, as observed in Southern Brazil;
(6) urbanization of rural areas, where neighborhoods are established near forests or rural areas. The most common example occurred in the southeastern part of the State of Minas Gerais, where there was an invasion of rural dwellings by rats from an inundated floodplain during a period of torrential rains, in addition to other factors that have not been fully investigated, highlighting the great complexity of HPS epidemiology in Brazil.

Another interesting factor for Brazil is the occurrence of a natural phenomenon called “ratada”, which refers to an increase in the rodent population due to a greater supply of seeds produced during the cyclical blooming and fruiting of certain bamboo species native to the Atlantic Forest. At the end of the bamboo cycle, the reduction in available natural food supply (seeds) combined with inadequate storage of animal feed inside human dwellings act as attractants for rodents that migrate from the forest to inside houses [266]. This phenomenon has been linked to some cases in the states of the Atlantic Forest in Southern Brazil [267].

Of the various bamboo species in Southern Brazil, “taquara lixa” (*Merostachys fistulosa*) is especially important. The likely cycle of taquara lixa varies between 31, 32 and 34 years, and considering the reports from inhabitants of various municipalities in the region, the last confirmed “ratada” in the State of Santa Catarina occurred in 1972. Thus, during the period from 2004 to 2005 (reinforcing the cyclic nature of bamboo), a new bamboo bloom occurred in Santa Catarina, which drew greater attention from the epidemiological surveillance team from the state due to the possible occurrence of “ratadas” in flowering areas [267]. Interestingly, only small foci were observed, without the alarming levels observed in 1972. In addition to a smaller area covered by this native bamboo species, other less productive species (less seed production) that do not produce “ratadas” may be taking the place of taquara lixa [268,269]. Little scientific information is available regarding the phenomenon of “ratadas” associated with bamboo flowering in Brazil. An ecological study on the population dynamics of rodent hantavirus reservoirs in southern Brazil between 2004 and 2006 found an increase in the populations of *O. nigripes* and *Thaptomys nigrita* due to the flowering and fruiting of bamboos in the region [270].

In summary, the main factor correlated with human epidemics or small outbreaks of hantavirus appears to be an increase in rodent density, which promotes efficient propagation of the virus among the rodent population and a subsequent increase in the number of human cases. However, a simple increase in host rodent population density does not necessarily result in an increase in viral infection among the human population. The human activity behavior, the landscape use and the type of circulating hantavirus are also important factors for human infection confirming the complex set of interactions.

Given the complexity of hantavirus reservoir status and transmission, long-term ecological studies are needed to better understand the factors responsible for the oscillation in human cases on the local, regional and continental scales.

## 4. Conclusions

Since the early 1990s, when a new syndrome caused by the viral genus *Hantavirus* was identified, more than 80 novel hantaviruses genotypes have been identified around the World. Several factors can explain the increasing number of hantaviruses identified, including greater interest from the scientific community, improvements in the eco-epidemiological monitoring infrastructure by the surveillance system due to high lethality of hantavirus infections (especially for HPS) and an increase in the availability of diagnostic techniques for detection and characterization of viruses and their animal reservoirs, among others.

Given the discovery of phylogenetically divergent hantaviruses in various insectivorous bat species distributed across distinct geographical regions on four continents, a distinct possibility exists that rodents are not the primary reservoirs for hantaviruses. Moreover, other mammals with a common ancestry or that share ecosystems with Talpidae and Soricidae can serve as reservoirs and may have been important in the evolutionary history and diversity of hantaviruses. New hantaviruses, new mammalian host species and new human syndromes associated with hantaviruses are expected to be discovered in the near future. This possibility is strengthened by the recent discovery of bats that are known to host a wide range of emerging pathogens, including other viruses in the *Bunyaviridae* family, in addition to the great diversity of bat species, their flight abilities and their vast geographical distribution.

Despite the growing number of studies on hantaviruses in rodents, bats, shrews and moles, the results of research with respect to the presence of hantavirus infections in other wild (non-human primates) and domestic (dogs, cats and pigs) animal species have only shown serological evidence of infection based on the presence of IgG antibodies but not based on the direct detection of hantavirus RNA. Given that all other genera of the family *Bunyaviridae* are transmitted by arthropods, a study recently investigated the presence of hantavirus in ticks, but no viral RNA was detected [271,272]. Although studies on hantaviruses in various animal species are continually conducted, the results obtained to date support the idea that the participation of non-human primates, domestic animals and ticks in hantavirus transmission is highly unlikely.

Considering that Brazil is biologically rich containing one of the most diverse continental biotas [273], a significant number of novel hantaviruses could be identified in the near future, particularly in the Amazon and Atlantic Forest biomes.

Finally, the growing number of publications on hantaviruses in various vertebrate and invertebrate animal species has stimulated interest in identifying new reservoirs. The increase in popularity of hantavirus reservoir research increases the need to establish a consensus nomenclature for the newly identified hantaviruses and reinforces the importance of further ecological studies aimed at better understanding the dynamics of hantavirus transmission to human populations.

## Supplementary Files

Supplementary File 1 PDF-Document (PDF, 492 KB)
