# Supplementary material for: Hantavirus Reservoirs: Current Status with an Emphasis on Data from Brazil
Source: Viruses. 2014 Apr 29;6(5):1929–73. doi: 10.3390/v6051929 (PMC4036540; doi:10.3390/v6051929)
Supplement: Supplementary File 1 — PDF-Document (PDF, 492 KB) [file viruses-06-01929-s001.pdf]

Supplementary Table 1. Geographical distribution of hantaviruses associated with rodent reservoirs in the Old World.

Hantavirus	Human disease	Reservoirs (common name)	Known virus range	Genbank acession number of complete sequence			First references to known hosts
				S	M	L	
**Hantaviruses associated with rodents of the family Muridae, subfamily Murinae**							
Hantaan * (HTNV)	HFRS	*Apodemus agrarius* (Striped field mouse)	China; Korea; Japan;Russia	D25533	L08753	NC005222	[1]
Thailand * (THAIV)	ND	*Bandicota indica* (Great bandicoot rat)	Thailand	AM397664	L08756	-	[274]
Seoul * (SEOV)	HFRS	*Rattus rattus* (Black rat); *Rattus norvegicus* (Brown rat)	Belgium; Brazil; China; France; Ireland; Korea; Cambodia; Laos; Portugal; Russia; Singapore; Taiwan; United Kingdom; Vietnam	AF187082	AF18708	AF285266	[275]
Gou (GOUV)	ND	*Rattus rattus* (Black rat)	China	AF184988	AF145977	-	[276]
Da Bie Shan (DBSV)	ND	*Niviventer confucianus* (Chinese white-bellied rat)	China	AB027523	AB027115	DQ989237	[276]
Serang (SERV)	ND	*Rattus tanezumi* (Asian house rat)	Indonesia;China	-	-	-	[134]
Saaremaa * (SAAV)	HFRS	*Apodemus agrarius* (Striped field mouse)	Estonia; Finland; Germany; Hungary; Lithuania; Russia; Slovakia; Slovenia	AJ616854	-	AJ410618	[277]
Kurkino (KURV)	HFRS	*Apodemus agrarius* (Striped field mouse)	Estonia (mainland); Germany; Slovakia; Russia; Hungary; Slovenia; Croatia;	-	-	-	[278]
Sochi (SOCV)	HFRS	*Apodemus ponticus* (Black Sea field mouse)	Russia	JF920150	JF920149	JF920148	[279]
Dobrava-*Belgrade* * (DOBV)	HFRS	*Apodemus flavicollis* (Yellow-necked mouse)	Bulgaria; Croatia; Greece Hungary; Serbia and Montenegro; Slovakia; Slovenia	L41916	L33685	GU904045	[5]
Amur/ Soochong (ASV)	HFRS	*Apodemus peninsulae* (Korean field mouse)	China; Korea; Russia	AB071184	AB127994	AB620030	[280]
Sangassou * (SANGV)	ND	*Hylomyscus simus* (African wood mouse)	Africa	JQ082303	JQ082301	JQ082302	[281]
Tigray (TIGV)	ND	*Stenocephalemys albipes* (White-footed mouse)	Ethiopia	-	-	-	[282]

Supplementary Table 1. *Cont.*

Hantavirus	Human disease	Reservoirs (common name)	Known virus range	Genbank acession number of complete sequence			First references to known hosts
				S	M	L	
**Hantaviruses associated with rodents of the family Cricetidae, subfamily Arvicolinae**							
Puumala * (PUUV)	HFRS (NE)	*Myodes glareolus* (Bank vole)	Austria; Belgium; Bosnia and Herzegovina; Bulgaria; Czech Republic; Denmark; Estonia; Finland; France; Germany; Hungary; Ireland; Lithuania; Luxembourg; The Netherlands; Norway; Poland; Romania; Russia; Slovakia; Slovenia; Sweden; Turkey	NC_00522	NC_00522	M63194	[3]
Hokkaido (HOKV)	ND	*Myodes rufocanus* (Grey-sided vole)	Japan	AB675480	AB675451	AB712372	[283]
Muju (MUJV)	ND	*Myodes regulus* (Royal vole)	Korea	DQ138142	EF198413	-	[24]
Tula * (TULV)	-	*Microtus arvalis* (European common vole)	Austria; Belgium; Croatia; Czech Republic; Finland; France; Germany; Hungary; Lithuania; The Netherlands; Poland; Russia; Serbia; Slovenia; Slovakia	Z49915	Z66538	NC_005226	[138]
Topografov * (TOPV)	ND	*Lemmus sibiricus* (Siberian brown lemming)	Palaearctic tundra	AJ01164	AJ011647	AJ011649	[284]
Khabarovsk (KHAV)	ND	*Microtus maximowiczii* (Maximowicz's vole)	China;Russia	U35255	AJ011648	-	[151]
Vladivostok (VLAV)	ND	*Microtus fortis* (Reed vole)	China;Russia	-	-	-	[285]
Yuanjiang (YUJV)	ND	*Microtus fortis* (Reed vole)	China	FJ170795	-	-	[286]
Luxi (LUXV)	HFRS	*Eothenomys miletus* (Yunnan red-backed vole)	China	-	-	-	[287]
Tatenale (TATV)	ND	*Microtus agrestis* (Grey-brown vole)	United Kingdom	-	-	-	[288]

HFRS: Haemorraghic Fever with Renal Syndrome; ND: none documented; NE: epidemic nephropathy; * Officially recognised as viral species by the ICTV; – Not available in GenBank.

Supplementary Table 2. Geographical distribution of hantaviruses associated with rodent reservoirs in the New World.

Hantavirus	Human disease	Reservoirs (common name)	Known virus range	Genbank acession number of complete sequences S	M	L	First reference to known hosts
**Hantaviruses associated with rodents of the family Cricetidae, subfamily Sigmodontinae**							
Anajatuba (ANAJV)	HPS	*Oligoryzomys fornesi (Fornes´ colilargo)*	Northern Brazil	-	-	-	[171]
Andes (ANDV) *	HPS	*Oligoryzomys longicaudatus (Long-tailed pygmy rice rat)*	Southwestern Argentina and Chile	AF324902 [b]	AF324901 [b]	AF291704 [b]	[169]
Alto Paraguay (ALPV)	ND	*Holochilus chacarius (Chacoan marsh rat)*	Western Paraguay	DQ345762	-	-	[175]
Ape Aime-Itapua (AAIV) [a]	ND	*Akodon montensis (Montane akodont)*	Eastern Paraguay	-	-	-	[175]
Araraquara (ARAV)	HPS	*Necromys lasiurus (Hairy-tailed bolo mouse)*	Eastern Brazil	EF571895[b]	-	-	[200]
Bayou (BAYV) *	HPS	*Oryzomys palustris (Marsh rice rat)*	Southeastern USA	GQ200820	GQ244521	GQ244526	[289]
Bermejo (BERV)	ND	*Oligoryzomys chacoensis (Chacoan pygmy rice rat)*	Northwestern Argentina	AF482713	-	-	[169]
Black Creek Canal (BCCV) *	HPS	*Sigmodon hispidus (Hispid cotton rat)*	Southern Florida, USA; Venezuela; Peru	L39949	L39950	-	[290]
Calabazo (CALV)	ND	*Zygodontomys brevicauda (Short-tailed cane mouse)*	central-western Panamá; Northwestern Colombia	-	-	-	[291]
Caño Delgadito (CADV) *	ND	*Sigmodon alstoni (Alston´s cotton rat)*	Western Venezuela	DQ285566	DQ284451	GQ200821	[292]
Castelo dos Sonhos (CASV)	HPS	*Oligoryzomys utiaritensis (No comom name)*	Central-western Brazil	JX443691	-	-	[201]
Catacamas (CATV)	ND	*Oryzomys couesi (Coues' rice rat)*	Eastern Honduras	DQ256126	DQ177347	FJ858378	[293]
Central Plata	HPS	*Oligoryzomys flavescens (Yellow pygmy rice rat)*	Southern Uruguay	-	-	-	[192]
Choclo (CHOV)	HPS	*Oligoryzomys fulvescens (Fulvous pygmy rice rat)*	Central-western Panama	DQ285046	DQ285047	EF397003	[290]
Itapua (ITAV)	ND	*Oligoryzomys nigripes (Black-footed pygmy rice rat)*	Eastern Paraguay	DQ345765	-	-	[175]
Jabora (JABV)	ND	*Akodon montensis (Montane akodont)*	Southern Brazil; Eastern Paraguay	JN232078	-	-	[177]
Juquitiba (JUQV)	HPS	*Oligoryzomys nigripes (Black-footed pygmy rice rat)*	Southern Brazil; Eastern Paraguay; Northeastern Argentina; Uruguay	KC422344	FJ409556 [b,c]	-	[200]

Supplementary Table 2. *Cont.*

Hantavirus	Human disease	Reservoirs (common name)	Known virus range	Genbank acession number of complete sequences			First reference to known hosts
				S	M	L	
Laguna Negra (LANV) *	HPS	*Calomys laucha (Small vesper mouse)*	Western Paraguay	AF005727	AF005728	-	[173]
		Calomys callosus (Large vesper mouse)	Bolivia; Northwestern Argentina	-	-	-	[179]
		Callomys callidus (Crafty vesper mouse)	Midwest Brazil	-	-	-	[174]
Lechiguanas (LECV)	HPS	*Oligoryzomys flavescens (Yellow pygmy rice rat)*	Central Argentina	AF482714	AF028022	-	[294]
Maciel (MACV)	ND	*Necromys obscurus (Dark bolo mouse)*	Central Argentina	AF482716	-	-	[294]
Maporal (MAPV)	ND	*Oligoryzomys delicatus (Delicate pygmy rice rat)*	Western Venezuela	AY267347	AY363179	EU788002	[295]
Muleshoe (MULV) *	ND	*Sigmodon hispidus (Hispid cotton rat)*	Texas to Southern Nebraska, USA	U54575	-	-	[296]
Oran (ORNV)	HPS	*Oligoryzomys longicaudatus (Long-tailed pygmy rice rat)*	Northwestern Argentina	AF482715	AF028024		[169]
Pergamino (PERV)	ND	*Akodon azarae (Grass field mouse)*	Central Argentina	AF482717	-	-	[293]
Playa de Oro (OROV)	ND	*Oryzomys couesi (Coues' rice rat)*	Colima, Western México	EF534079	-	-	[297]
Rio Mamore (RIOMV)*	HPS	*Oligoryzomys microtis (Small-eared pygmy rice rat)*	Bolivia; Northeastern Peru; Northwestern Brazil	U52136	FJ608550	FJ809772	[298]
Rio Mearim (RIMEV)	ND	*Holochilus sciureus (Marsh rat)*	Northeastern Brazil	-	-	-	[171]

Supplementary Table 2. *Cont.*

Hantavirus	Human disease	Reservoirs (common name)	Known virus range	Genbank acession number of complete sequences S	M	L	First reference to known hosts
**Hantaviruses associated with rodents of the family Cricetidae, subfamily Neotominae**							
Sin Nombre (SNV) *	HPS	*Peromyscus maniculatus (Deer mouse)*	North USA; Canada; Mexico	L37904	L37903	L37901	[9]
Blue River (BLUV)	ND	*Peromyscus leucopus (White-footed mouse)*	Central USA	-	AF030551	-	[42]
New York (NYV) *	HPS	*Peromyscus leucopus (White-footed mouse)*	East and central USA	U09488[b]	U36802	-	[165]
El Moro Canyon (ELMCV) *	ND	*Reithrodotomys megalotis (Western harvest mouse)*	Western USA; México	U11427	U26828	-	[299]
Rio Segundo (RIOSV) *	ND	*Reithrodontomys mexicanus (Mexican harvest mouse)*	Costa Rica	U18100	-	-	[300]
Limestone canyon (LSCV)	ND	*Peromyscus boylii (Brush mouse)*	Southwestern USA	-	-	-	[181]
Monongahela (MGLV)	HPS	*Peromyscus maniculatus (Deer Mouse)*	Eastern USA; Canada	U32591	-	-	[166]
Montano (MTNV)	ND	*Peromyscus beatae (Orizaba deer mouse)*	México	AB620100	AB620101	AB620102	[167]
Carrizal (CARV)	ND	*Reithrodontomys sumichrasti (Sumichrast´s harvest mouse)*	México	AB620103	AB620104	AB620105	[167]
Huitzilac (HUIV)	ND	*Reithrodontomys megalotis (Western harvest mouse)*	México	AB620106	AB620107	AB620108	[167]
**Hantaviruses associated with rodents of the family Cricetidae, subfamily Arvicolinae**							
Prospect Hill (PHV)	ND	*Microtus pennsylvanicus (Meadow vole)*	USA; Canada	U47136	X55129	EF646763	[6]
Bloodland Lake (BLLV)	ND	*Microtus ochrogaster (Prairie vole)*	USA	U19303	-	-	[301]
Isla Vista (ISLAV)	ND	*Microtus californicus (California vole)*	USA	IVU31534	-	-	[302]

HPS: Hantavirus Pulmonary Syndrome; ND: none documented; * Officially recognised as viral species by the ICTV; – Not available in GenBank; [a] The AAIV genotype was a reassortment of the S-segment of the JABV-like viral genotypes and the M-segment of the AND-like viral genotypes [186]. [b] Sequence from human case. [c] Sequence of Araucaria virus, a variant of JUQV. ** *Oligoryzomys longicaudatus* distribution does not include the Northwestern Argentina where the hantavirus Oran was identified in *Oligoryzomys* specimens. *Oligoryzomys brendae* is the only large-sized species of *Oligoryzomys* inhabiting the northwestern Argentina [303], and, probably, this species is the reservoir of hantavirus Oran according to this new taxonomic arrangement or *O. chacoensis* as recently described by Rivera and colleagues [188].

Supplementary Table 3. Geographical distribution of hantaviruses associated with insectivorous reservoirs and chiropterans in the Old and New World.

Hantavirus	Reservoirs (common name)	Known virus range	Genbank acession number of complete sequences			First references to known hosts
			S	M	L	
**Hantaviruses associated with shrews and moles of the families Soricidae and Talpidae**						
Thottapalayam (TPMV) *	*Suncus murinus* (Asian house shrew)	India; China; Nephal	AY526097	DQ825771	DQ825770	[20]
Imjin (MJNV)	*Crocidura lasiura* (Ussuri white-toothed Shrew)	South Korea	EF641804	EF641797	EF641806	[221]
Nova (NVAV)	*Talpa europaea* (European common mole)	South-western Hungary; France	FJ539168	-	FJ593498	[212]
Asama (ASAV)	*Urotrichus talpoides* (Japanese shrew mole)	Japan	EU929070	EU929073	EU929076	[26]
Seewis (SWSV)	*Sorex araneus* (Eurasian common shrew)	European Russia; Siberia; Belgium; Yugoslavia; Hungary; Finland Austria; Czech Republic; Slovakia; Germany	EF636024	-	-	[23]
Oxbow (OXBV)	*Neurotrichus gibbsii* (American shrew mole)	Oregon, USA	FJ539166	FJ539167	-	[27]
Cao Bang (CBNV)	*Anourosorex squamipes* (Chinese shrew mole)	Vietnam	EF543524	EF543526	EF543525	[24]
Camp Ripley (RPLV)	*Blarina brevicauda* (Short-tailed shrew)	Minnesota, USA	-	-	-	[25]
Tanganya (TGNV)	*Crocidura theresae* (Therese's shrew)	Guinea	-	-	-	[22]
Jemez Springs (JMSV)	*Sorex monticolus* (dusky shrew)	New Mexico; Colorado, USA	FJ686859	-	-	[222]
Ash River (ARRV)	*Sorex cinereus* (masked shrew)	Minnesota, USA	-	-	-	[222]
Kenkeme (KKMV)	*Sorex roboratus* (flat-skulled shrew)	Sakha Republic in Eastern Siberia	GQ306148	-	-	[28]
Rockport (RKPV)	*Scalopus aquaticus* (Eastern mole)	Texas, USA	HM015218	HM015219	HM015220	[29]
Asikkala (ASIV)	*Sorex minutus* (Eurasian pygmy shrew)	Czech Republic; Germany; Filand	KC880342	KC880345	-	[304]
Azagny (AZGV)	*Crocidura obscurior* (West African pygmy shrew)	Coast of Côte d'Ivoire	-	-	-	[305]

Supplementary Table 3. *Cont.*

Hantavirus	Reservoirs (common name)	Known virus range	Genbank acession number of complete sequences			First references to known hosts
			S	M	L	
Laihia (LAIV)	*Neomys fodiens* (water shrew)	Finland	-	-	-	[81]
Jeju (JEJV)	*Crocidura shantungensis* (Asian lesser white-toothed shrews)	Jeju Island Southern coast of Korea	HQ834695	HQ834696	HQ834697	[225]
Boginia (BOGV)	*Neomys fodiens* (Eurasian water shrew)	Central Poland	-	-	-	[306]
Yakeshi (YKSV)	*Sorex isodon* (Taiga shrew)	Eastern China	JX465423	JX465403	-	[226]
Lianghe (LHEV)	*Anourosorex squamipes* (Chinese mole shrew)	Southwest China	JX465406	JX465390	-	[226]
Bowé (BOWV)	*Crocidura douceti* (Doucet's musk shrew)	Southwestern Guinea	KC631782	KC631783	KC631784	[306]
**Hantaviruses associated with bats of the families Vespertilionidae, Nycteridae and Rhinolophidae**						
Mouyassue (MOUV)	*Neoromicia nanus* (banana pipistrelles)	Southeastern region of Côte d'Ivoire	-	-	-	[30]
Magboi (MGBV)	*Nycteris hispida* (hairy slit-faced)	Sierra Leone, África	-	-	-	[31]
Xuan Son (XSV)	*Hipposideros pomona* (Pomona roundleaf)	Vietnam	-	-	-	[227]
Huangpi (HUPV)	*Pipistrellus abramus (japonese house or japonese pipistrelle)*	China	JX473273	-	-	[226]
Longquan (LQUV)	*Rhinolophus affinis* (Intermediate Horseshoe) *Rhinolophus sinicus* (Chinese Rufous Horseshoe) *Rhinolophus monoceros* (Formosan Lesser Horseshoe)	China	JX465413	JX465398	-	[226]

* Officially recognised as viral species by the ICTV; – Not available in GenBank.
